# CircRNAs—Potential Diagnostic Biomarkers and Therapeutic Targets for Receptive and Cancerous Endometrium

**DOI:** 10.3390/epigenomes9040047

**Published:** 2025-11-17

**Authors:** Antoan Milov, Maria Nikolova, Stoilka Mandadzhieva, Nina Doncheva, Nadezhda Milova, Angel Yordanov

**Affiliations:** 1 Center for Women’s Health, 4000 Plovdiv, Bulgaria; antoan.milov@cwh-bg.com (A.M.);; 2 Faculty of Medicine, Medical University of Plovdiv, 4000 Plovdiv, Bulgaria; 3 Pathophysiology Department, Medical University of Plovdiv, 4000 Plovdiv, Bulgaria; 4 Department of Pharmacology, Toxicology and Pharmacotherapy, Medical University of Plovdiv, 4000 Plovdiv, Bulgaria; 5 Department of Gynecological Oncology, Medical University Pleven, 5800 Pleven, Bulgaria

**Keywords:** circRNAs, miRNAs, endometrial receptivity, endometrial cancer, epigenetic regulation, target therapy, molecular diagnostics

## Abstract

Circular RNAs (circRNAs) are small, non-coding RNAs in which the 5′ and 3′ ends are linked covalently by back-splicing of exons from a single pre-mRNA. More and more scientific evidence is gathered for their wide distribution in the animal world, playing the role of regulators for biological processes, being cell- and tissue-specific. They can influence cellular physiology by various molecular mechanisms, finally modulating gene expression. CircRNAs are believed nowadays to be expressed in both receptive and cancerous endometrium. Due to their abundant expression in the endometrial tissue and their small size and stability, they have been considered potential diagnostic markers and treatment targets for endometrial-related diseases. The regulation of proliferation and differentiation is essential for the formation of receptive endometrium and for endometrial cancer emergence and progression. The receptive endometrium can be regarded as the most highly differentiated state of the endometrium. In contrast, the cancerous endometrium is characterized by a high level of proliferation and the lowest degree of differentiation. These endometria could be conditionally considered opposites. We are investigating the circRNA–miRNA–mRNA regulatory networks that can promote or suppress the proliferation and differentiation of endometrial cells by activating specific signaling pathways in both receptive and cancerous endometria. It could be worth knowing whether there are universal endometrial switches responsible for proliferation and differentiation processes that can alter the balance between them. We are interested in their clinical application as biomarkers and therapeutic targets for both endometrial receptivity issues and EC cases, particularly in diagnosis, progression assessment, and outcome prediction.

## 1. Introduction

Circular RNAs (circRNAs) are closed non-coding RNAs in which the 5′ and 3′ ends are linked covalently by back-splicing of exons from a single pre-mRNA. Increasing evidence has been gathered for the wide expression of circRNAs in mammalian cells, cell-type- and tissue-specific. circRNAs clearly participate in cellular physiology through various molecular mechanisms, modulating gene expression or translation of gene regulators. The biogenesis of circRNAs is regulated by *cis*-elements and *trans*-factors. They exert control of gene expression, both cell- and tissue-dependent [1].

**Figure 1:**
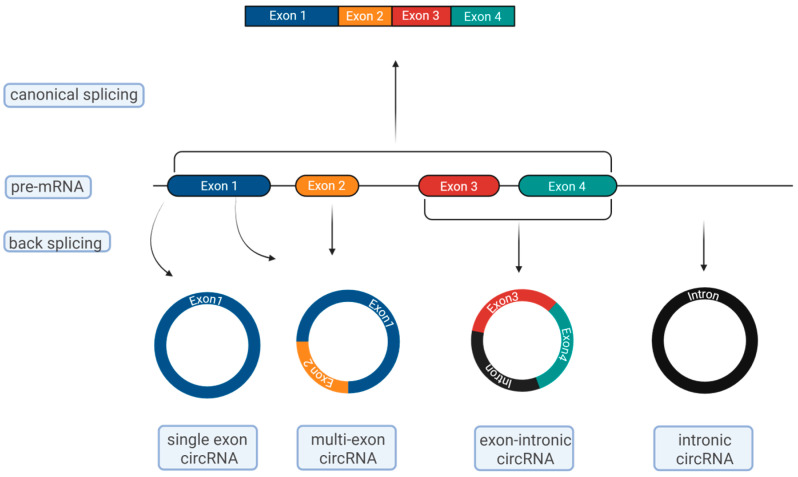
Biogenesis of circular RNAs (created in Biorender. Milov, A. (2025) https://BioRender.com/3cbbs9g).

Most eukaryotic genes are interrupted by non-coding introns. The introns are removed by splicing in the process of RNA formation, resulting in the fusion of exons in linear transcripts (Figure 1). By splicing, several isoforms can also be generated from a given gene that can exert different functions, be located in different cell types, and play different roles. More than 90% of human genes have isoforms [2].

Circular RNAs are formed by a back-splicing mechanism, in which the 5′ end of the upstream exon is non-collinearly spliced with the 3′ end of the downstream exon; thus, the formed circular molecules have no 5′ cap and 3′ tail, which makes them resistant to RNases in the cytoplasm compared to the linear species [3]. With the advance of next-generation sequencing data gathering, there is evidence that the circRNA expression profiles can be higher than the linear ones and that many of them are conserved across species [4]. This growing data of more and more newly discovered circRNA molecules has been imported into several databases—circBase, exoRBase, circNet, etc.—in order to curate different species of circRNAs, circRNAs in different cell types, and circRNAs in health and disease [5,6]. The generation of such databases creates opportunities for better exploration, understanding, and comparison of the multiple functions of the circRNAs.

Circular RNAs are spliced from coding pre-mRNAs but are considered long non-coding RNAs and regulators of gene expression. In their interaction with miRNAs, they play the role of a “sponge,” harboring several miRNA binding sites. On the other hand, they can form complexes with proteins and participate in the cell cycle or in translation, or act as intercellular signaling molecules, included in exosomes [7]. Still, there are many uncertainties about the biogenesis of circRNAs, their functions in cells, the regulatory networks they participate in, and the control of regulatory networks.

CircRNAs are considered specific for the different cell types. We aim to analyze the expression profile data of circRNAs in both receptive and cancerous endometria. These two types of endometrium can conditionally can be considered antipodes: the receptive endometrium being the most highly differentiated and the cancerous endometrium having a high level of proliferation and the lowest differentiation. In this review, we aim to investigate whether the circRNA/miRNA/mRNA networks, by activating specific signaling pathways in both receptive and cancerous endometria, can promote or suppress the proliferation and differentiation of endometrial cells. It could be essential to find if there are universal endometrial switches that could alter the equilibrium between proliferation and differentiation processes. CircRNAs have the potential for clinical application as biomarkers and therapeutic targets for both problematic endometrial receptivity cases and EC (endometrial cancer) cases in diagnosis, treatment, follow-up and prognosis.

## 2. Biogenesis of circRNAs

Circular RNAs are formed by back-splicing, as a result of which the 5′ end of the upstream exon is non-collinearly spliced with the 3′ end of the downstream exon. The back-splicing is regulated by *cis-*(DNA sequences) and *trans-*(RNA binding proteins) factors. Most circular RNAs contain more than one exon with flanking introns, but in cases when the exon is only one, it is a much longer exon (when a circular RNA is formed exclusively of exons, it is called ecircRNA); in addition, a circRNA can consist of both exons and introns (EIcircRNA) or of introns only (ciRNA) (Figure 1) [8]. There are two major proposed models of circRNa biogenesis: direct back-splicing and lariat-driven circularization. During direct back-splicing, the splicing sites are brought close to each other by complementary base pairing of inverted repeats in introns flanking the exons to be circularized. After circularization, the introns are removed, and ecircRNA is formed, or in case they remain, EIcircRNA is formed. As an alternative, circRNAs can be achieved through lariat-driven circularization. EcircRNAs, EIcircRNAs and ciRNAs can be generated by this mechanism, and ciRNAs can occur from introns removed during pre-mRNA splicing. Usually, these introns are removed and degraded, but the ones containing 7nt GU-rich element, close to the 5′ end, and 11nt C-rich element, close to the branching point, are retained, and thus ciRNas are formed. Most of the circRNAs are ecircRNAs and are found in the cytoplasm, whereas EIcircRNas and ciRNAs are located in the nucleus. All the cumulated data by now suggests that there is no specific exon sequence responsible for circRNA formation [9].

It is interesting to mention that the flanking introns of the circRNAs are longer than the average and contain a lot of complementary sequence repeats [10]. These complimentary flanking sequences were described first in mice, the circular *SRY* gene, suggesting that the complementary intronic sequences (CIS) have an impact on the formation of the circular RNAs [11]. It was observed that the deletion of CIS leads to the abolishment of some circRNAs [12]. Some Alu repetitive elements were demonstrated to be enriched in flanking introns of human circRNAs [13] and the complementary pairing between Alu elements with reverse orientation can lead to an alternative expression of linear or circular isoforms [14,15].

Trans factors in circRNA formation have also been studied. Mbl binding sites on flanking introns are necessary for circMbl formation, and the increased expression of Mbl binding sites enhances circMbl expression; hence, the splicing factor is suspected to be important for the biogenesis of circRNA [9]. Some authors have demonstrated that the disruption of splicing factor QKI or its binding sites on flanking introns decreases the generation of circRNA during EMT (epithelial–mesenchymal transformation) [16]. Splicing factors can both up- and down-regulate circRNA formation [12]. For example, the splicing factor ESRP1 can promote the expression of *circBIRC6* [17], while the disruption of splicing of Hrb27C increases the expression of circular laccase2 in Drosophila. Some binding proteins can also regulate circRNA formation—ILF3 downregulates circRNA expression ILF3, while DHX9 has been shown to upregulate the expression of circRNAs in humans [18].

## 3. Mode of Action of circRNAs

**Figure 2:**
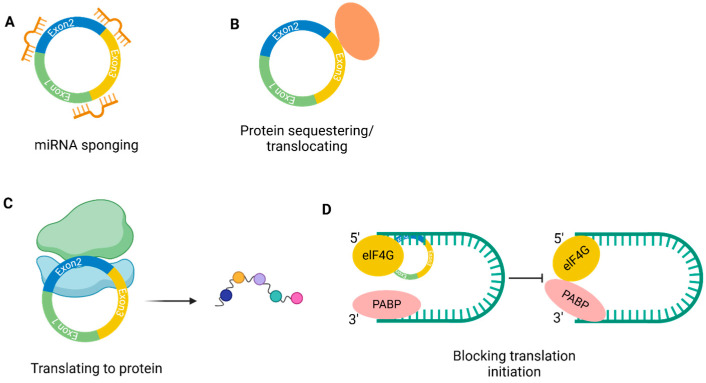
Mode of action of circRNAs. (**A**). MiRNA sponging mechanism, (**B**). Protein sequestring, (**C**). CircRNA translating into proteins, (**D**). CircRNAs blocking translation initiation (Created in Biorender. Nikolova, M. (2025) https://BioRender.com/ogqqz22).

CircRNAs majorly act by inhibiting miRNA activity. They serve as a sponge for miRNAs, and finally regulate target gene expression (Figure 2A). There are several sites in the circular sequence that can bind one or multiple miRNAs. Thus the first identified circRNA, the human *CDR1as*, has several binding sites for miRNA-7, 63 of which are genetically conserved in other species. The knocking out of *CDR1as* in zebrafish and mice alters the expression of miRNA-7 and leads to midbrain impairment, whereas in humans it affects cell proliferation, insulin secretion, and pathobiology of the myocardial infarction [19,20,21]. This genetic conservatism has been used in studies to create a transgenic sponge model [22]. CircRNAs regulate the expression of miRNAs and thus regulate different downstream genes. It has been found that *circITCH* sequesters miR-214 and miR-22-3p and thus upregulates ITCH and CBL expression, which participate in the regulation of the WNT/β-catenin pathway [23,24].

Besides interacting with miRNAs, circRNAs can serve as *protein baits* and alter cellular function. *CircFOXO3* is shown to trap CDK2/p21 and HIF-a/ID1 in the cytoplasm, blocking cell cycle progression and inducing cell aging, respectively [25]. In breast cancer cells, *circDNMT1* activates autophagy by causing P53 and AUF1 nuclear translocation [26] (Figure 2B).

Circular RNAs are considered with low coding potential, but circRNAs have been found as well in the ribosome entry site. It has been proven that N^6^-methyladenosine modification can be *translated into peptides* both in vitro and in vivo [27] as it contains the start codon of cognate mRNAs and associates with ribosomes [28]. *CircZNF609* regulates myogenesis and can be translated into peptides, suggesting that *circZNF609* may exert its function through protein expression [29]. *LncRNA-PINT* is translated into a small peptide that can suppress glioblastoma cell proliferation, which is mediated by the hindering of PAF1c and halting translational elongation of oncogenes [30] (Figure 2C). CirRNA can directly bind its parent mRNA and *regulate its translation* as well, and the interaction between them depends on primary sequence and the tertiary structures [30] (Figure 2D).

## 4. Regulatory Networks and the Control of Regulatory Networks

### 4.1. Regulation of Cell Proliferation by circRNAs

More and more circRNAs have been cited to regulate proliferation by affecting signaling pathways and transcription factors [31]. Several pathways regulate proliferation, but the WNT/β-catenin pathway has been described by many authors to regulate endothelial and endometrial proliferation [32,33]. There is evidence that *circHIPK3* can promote proliferation in human cell lines, probably by upregulation of IL6R expression [34]. Transcription factors can also be targets of circRNA regulation. For example, disruption of *circTCF25* in cancer cells downregulates CDK6 expression, promoting the proliferation of bladder cancer [35]. Circular RNAs may act as proliferation inhibitors. Increased expression of *circITCH* and *circZFR* upregulates PTEN expression and thus inhibits the proliferation of bladder cancer and HCC (hepatocellular carcinoma) cells [36]. In addition, *circITCH* promotes ITCH and CBL expression, which inhibits cell proliferation by downregulating the WNT/β-catenin pathway [23]. In a similar manner, *hsa_circ_0002052* induces APC2 expression, which causes β-catenin degradation and inhibits osteosarcoma cell proliferation [37]. Obviously, circRNAs can regulate cell proliferation through different mechanisms.

### 4.2. Regulation of EMT and Cancer Progression by circRNAs

EMT is observed in early development, wound healing, and the stem cell reparative cycle. EMT is subjected to precise regulation so that the proper differentiated cells can find their proper place at the proper time. It is observed in the early stages of cancer and in cancer progression and invasion [38]. There is scientific evidence that TGF-β family ligands induce EMT. They stimulate the phosphorylation and nuclear translocation of R-SMADs and co-SMADs and activate SNAI, bHLH, and ZEB transcription factors [38]. The accumulated data suggests that circRNAs regulate the EMT process and thus contribute to cancer progression. *circMYLK* was found to act on the TGF-β signaling pathway by increasing TRAF4 expression in prostatic carcinoma cells to decrease degradation of the TGF-β receptor and activate EMT [39]. It has been shown as well that circRNAs can inhibit EMT. An example of this is the upregulation of TRIM33 caused by the action of *circSMAD2* and the following decreased expression of SMAD4, which blocks the TGF-β signaling pathway in HCC (hepatocellular carcinoma) cells [40].

### 4.3. Regulation of Pluripotency and Early Lineage Differentiation by circRNAs

Pluripotent stem cells can be embryonic or induced, and can differentiate into many cell types. CircRNAs play roles in the maintenance of pluripotency and in differentiation of cells. Disruption of *circBIRC6* and *circCOROC1* negatively affects the maintenance of pluripotency; on the other hand, the expression of *circBIRC6* and *circCORO1C* activates pluripotency reprogramming of induced pluripotent stem cells. There is evidence that *circBIRC6* inhibits the activity of miR-34a and miR-145, thus ceasing the downregulation of pluripotency transcription factors NANOG, OCT4, and SOX2 [41]. Furthermore, circRNAs are involved in somatic stem cell differentiation as well. *CDR1as* has been shown to regulate neural development in zebrafish and osteoblastic differentiation of periodontal ligament stem cells, while abnormal expression of *circFGFR4*, *circSVIL,* and *circZNF609* induces myoblast differentiation [42,43,44].

## 5. Endometrium—Cytology, Histology, Signaling Pathways

**Figure 3:**
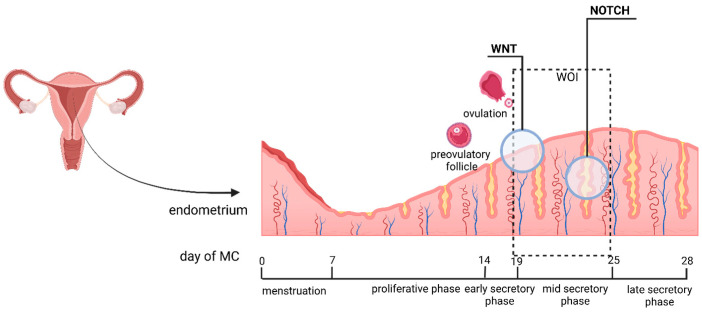
Temporal map of Wnt and NOTCH pathways in the endometrial cycle. (Created in Biorender Nikolova, M. (2025) https://BioRender.com/a6acgle).

The endometrium is the thick mucous membrane that covers the inner surface of the uterus, surrounded by a fibromuscular layer (myometrium) and an outer layer (serosa). Depending on its location in the uterus, the mucous membrane can be defined as the isthmic mucosa (or the mucosa of the lower uterine segment) and the corpus mucosa (the mucosa of the uterine body). The isthmic mucosa is a transitional zone between the endocervix and the corpus endometrium and is significantly thinner and poorly responsive to hormonal stimuli [45]. In the reproductive age, the endometrium of the body of the uterus undergoes cyclical changes under the influence of estradiol and progesterone. The endometrial cycle goes through several phases, menstruation, the proliferative phase, and the secretory phase, the latter subdivided into early secretory, mid-secretory, and late-secretory phases. The mid-secretory phase is considered the best time for implantation or the WOI (window of implantation) and has been referred to as the receptive phase, while the early secretory phase is referred to as the perceptive phase [46] (Figure 3).

The endometrium consists of a columnar epithelium forming numerous tubular glands that are supported by a vascularized stroma and could be divided into two layers—functional and basal [47].

The functional layer is a thick surface layer that exfoliates during menstruation and regenerates cyclically. It consists of three types of cylindrical epithelial cells—ciliary, lumenal, and secretory [48]. With each successive cycle, long, multifold, tubular glands are formed that open to the uterine cavity [46]. During the proliferative phase, when the functional layer grows, the endometrial glands have a relatively smooth outline, and numerous mitotic figures are present [46]. In the secretory phase, the endometrial glands are mature, with a folded contour and large vacuolar inclusions. A secretion is observed in the lumen of the glands. Nidation of the embryo takes place in the lumenal epithelium [48]. Hypotheses exist that at the transcriptional level, endometrial epithelial cells owe their differentiation to transcriptional factors, which regulate epithelial cell differentiation throughout the endometrial cycle. Differentiation of endometrial epithelial cells is associated with two signaling pathways—Wnt and NOTCH (Figure 3).

The Wnt signaling pathway is a conserved evolutionary pathway similar in many animals around the world [49]. The discovery of the Wnt signaling pathway was linked to research on oncogenic retroviruses. Infection of mice with murine mammary tumor virus led to the identification of a novel murine proto-oncogene, Int1 (integration 1) [49]. Later, it was reported that Int1 is highly conserved across multiple species, including humans and Drosophila, and that the Int1 gene is actually the already known and characterized Drosophila melanogaster Wingless (Wg) gene [49]. The Wnt signaling pathway regulates cell development, cell migration, cell polarity, neuronal differentiation, and organogenesis during embryonic development [50].

The NOTCH signaling pathway is also an ancient, evolutionarily conserved pathway. In 1914, Dexter [51] noticed the appearance of a notch in the wings of D. melanogaster—this historically became the reason for the name of the signaling pathway. In mammals, four NOTCH receptors are available—NOTCH1, NOTCH2, NOTCH3, and NOTCH4 [52]. This signaling pathway is activated in the process of ciliated cell differentiation in embryonic skin development [53], in neoangiogenesis [54], and in the differentiation of neurons in the brain, maintaining neuronal progenitor cells in a proliferative active state [55]. The Notch signaling pathway has an impact on determining the polarity of the embryo—it determines anterior–posterior polarity as well as left–right polarity.

Ciliary epithelial cells show high Wnt targeting activity, whereas secretory epithelial cells show high expression of transcription factors induced by Wnt inhibition or by NOTCH activation. From here comes the assumption about the role of NOTCH and Wnt in determining the type and function (differentiation into ciliary or secretory) of epithelial cells. Also, NOTCH expression is increased in secretory cells, while Wnt expression is more pronounced in lumenal and ciliary cells (Figure 2) [48].

### 5.1. Endometrium and Endometrial Receptivity

The endometrial lumenal epithelial cells are the first point of contact with the also polarized embryonic trophectoderm, and they would repel each other if the endometrial epithelium continued to maintain its polarity, rendering the endometrium unreceptive to the blastocyst attempting to implant. The endometrial lumenal epithelium loses its polarity during the mid-secretory phase of the MC (menstrual cycle) [56]. This makes the endometrium receptive. The loss of polarity is due to morphological and molecular changes occurring in the epithelial cells. Changes occur in microvilli, cell-membrane surface markers, intercellular junctions, and cytoskeletal molecules. To provide opportunities for invasion, the lumenal epithelium weakens the lateral connections between cells by reducing surface epithelial adhesion proteins and focal adhesion to the basal membrane [56]. All these changes can be summarized in the concept of plasma membrane transformation (PMT) [57], an important event for implantation to occur.

Abnormal endometrial receptivity is one of the main causes of embryo implantation failures and infertility. Any disturbance in the PMT affects endometrial receptivity and implantation. Parallels have been drawn between PMT and EMT in carcinogenesis [58]. PMT and EMT resemble each other in remodeling the actin cytoskeleton, integrin expression, and epithelial–stromal communication. Both processes are still being studied [58].

### 5.2. Repeated Implantation Failure (RIF) and circRNAs

One of the key functions of the endometrium is to implant and nourish the embryo to ensure pregnancy. Paradoxically, the endometrium cannot accept (implant) the embryo during most of the menstrual cycle, except for a narrow window of time called the WOI. Successful embryo implantation depends on synchronizing a viable embryo and a receptive endometrium.

In fact, insufficient uterine receptivity has been estimated to be the cause of two-thirds of failed implantations, while the embryo itself is responsible for one-third of them [59]. In patients with RIF, one out of four patients has been described with a temporal shift of the implantation window [60]. RIF has recently been found to be not only a matter of an asynchronous (shifted) implantation window but also a pathological (impaired) implantation window [61], suggesting the possibility that these women may be suffering from RIF of endometrial origin.

Impaired endometrial receptivity is considered to underlie infertility, infertility treatment failure, and recurrent pregnancy loss. In assisted reproductive technologies, where the best quality embryos are transferred to the uterus as standard, implantation failure remains an unsolved obstacle [62]. Therefore, a better understanding of endometrial receptivity and the importance of mechanisms involved in the functions of the mid-secretory endometrium is needed. Many studies have been conducted to identify genetic markers of receptive endometrium, few of which have real clinical use, and RIF therapeutic solutions are sought [63,64,65].

Circular RNAs (circRNAs) are important for various biological processes. The accumulated high-throughput data reveals that circRNAs are present in both normal and pathologically changed endometrium. They are abundant in expression and stability and small in size, which makes them potential diagnostic markers and treatment targets for endometrial-related diseases (like endometrial cancer) and dysfunctions (like endometrium-dependent repeated implantation failure). CircRNAs’ impact on the endometrium suggests further investigation in the future [47].

It has been shown that circRNAs regulate gene expression by sequestering miRNAs as miRNA sponges. CircRNA–miRNA–mRNA regulatory networks are formed [66,67]. Studies have been carried out to understand better endometrial receptivity by comparing DE circRNAs in PE (pre-receptive endometrium) and RE (receptive endometrium). A mechanism in which a circular RNA participates and sponges a miRNA, leading to alteration in receptivity, is cited [68]. CiR8073 was found to target three miRNAs (miR-181a, miR-449, and miR-34a) in caprine endometrial cells in vitro. This ciR8073 sponges miR-449a, which leads to increased expression of CEP55 (centrosomal protein55), and the axis ciR8073/miR449a/CEP55 contributes to endometrial receptor formation, and proliferation is activated in both receptive and cancerous endometrium in vitro [68].

Recent studies have revealed that abnormal blood flow influences endometrial receptivity and is considered a risk factor for RIF [64]. The vascularization flow index (VFI) on the day of ET (embryo transfer) is a factor affecting the implantation rate and maintenance of pregnancy [65]. Some circRNAs, acting as miRNA sponges, can affect endometrial angiogenesis [69]. Researchers have selected circRNA, miRNA, and mRNA datasets from the GEO (https://www.ncbi.nlm.nih.gov/geo/ (accessed on 10 August 2025)) database to screen out DEcircRNAs, DEmiRNAs and DEmRNAs between RIF patients and thereof predict angiogenesis-related DEmRNAs and angiogenesis-related circRNA–miRNA pairs and construct circRNA–miRNA–mRNA networks related to angiogenesis, incorporating Gene Ontology (GO) analysis and Kyoto Encyclopedia of Genes and Genomes (KEGG) pathway enrichment analysis. An experiment was carried out with a mouse model of RIF, based on the genetic conservativeness, and transcription levels of circRNAs, miRNAs, and mRNAs were detected to validate the achieved data [70]. This study obtained a circRNA–miRNA–mRNA regulatory network covering 45DEmRNAs, 10 DEmiRNAs, and 8 DEcircRNAs, co-expressed with angiogenesis. Six genes with an impact on angiogenesis were identified, among which were vascular endothelial growth factor A (VEGFA) and hypoxia-inducible factor 1 subunit alpha (HIF1A) (Table 1).

**Figure 4:**
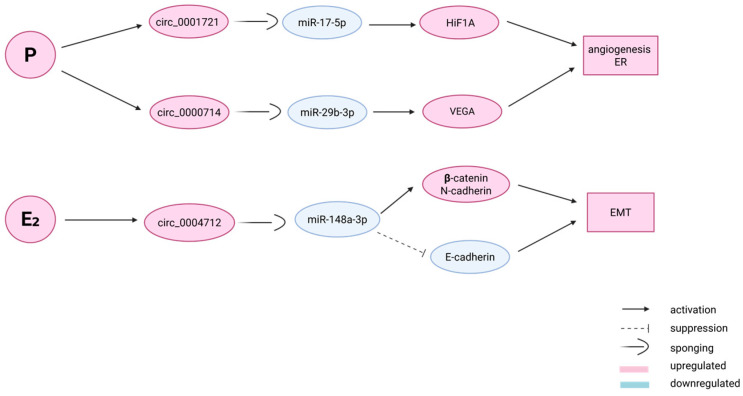
RIF and circRNAs. Upper two lines: P (progestogen) application’s impact on circ_0001721 and circ_0000714 expression levels (up-regulated) and the corresponding circRNA/miRNA/gene network resulting in promotion of angiogenesis and ER (endometrial receptivity). Bottom line: E_2_ (estradiol) application and the circRNA/miRNA/gene network promoting EMT.(Created in BioRender. Nikolova, M. (2025) https://BioRender.com/hvjvkcm).

Progesterone administration enhances endometrial angiogenesis through VEGF protein upregulation [69]. HIF-1 induces upregulation of miR-20a, which causes downregulation of dual specific phosphatase-2 (DSP-2), leading to the expression of several angiogenic genes [70,71]. A number of angiogenic genes were reported to be dysregulated in endometrial cancer (EC) and also play a key role in regulating embryo implantation. Some authors report that estrogen induces epithelial–mesenchymal transition via circ 0004712/miR-148a-3 sponge regulation [72,73]. Through this attempt to generate a circRNA–miRNA–mRNA regulatory network, the most promising factors for influence on the pathogenesis of angiogenesis related to endometrial RIF were found to be circRNA_0001721/miR-17-5p/HIF1A and circRNA_0000714/miR-29b-3p/VEGA axes (Figure 4) [68].

## 6. Endometrial Cancer

EC is cited to be the most common gynecologic cancer and among the six most common malignancies in women, together with breast, lung, colorectal, cervix, and thyroid cancers [74]. It occurs in endometrial tissue, most often in perimenopausal and postmenopausal women, and a trend has emerged of the manifestation of EC in younger women. It is believed that obesity, lack of physical activity, stress, and arterial hypertension are factors for this increase in incidence and appearance in younger age. However, risk factors like metabolic syndrome, exposure to estrogens, genetic predisposition, and longer life expectancy were also considered [75,76]. Postmenopausal bleeding is most often the first symptom to occur. Intermenstrual bleeding in younger women can be a suspicious sign. Endometrial biopsy and histological examination of the biopsy material are performed for the diagnosis. Although, in recent years, minimally invasive procedures have been used, still D&C (dilatation and curettage) is a diagnostic standard, and it should be taken in mind that in these situations, anesthesia and hospitalization are a must. New, non-invasive biomarkers for diagnosis and follow-up could benefit patients using a more easily accessible and reliable method [77].

FIGO (International Federation of Gynecology and Obstetrics) published in 2009 a classification of endometrial cancer. It was updated in 2023 with new, non-anatomical parameters for stage assessment [78], which makes EC staging more accurate and personalized and creates opportunities for more precise therapies [79]. The Cancer Genome Atlas was published in 2013 and provides information on the biological molecular features of EC. In accordance with it, there are four categories: ultra-mutated *POLE*, hypermutation/high microsatellite instability, high copy number, and low copy number of somatic varieties [80]. *Mutations in the POLE gene occur* in around 10% of all ECs. They are morphologically heterogeneous and affect young patients with normal BMI (Body Mass Index). This EC variant is said to have a more favorable prognosis and sensitivity to adjuvant therapy [81]. DNA polymerase-ε is believed to participate in the synthesis of the leading strand during DNA replication and also plays a role in repairing and correcting newly synthesized DNA strands [81]. Mutations in the epsilon polymerase lead to impaired 3′ to 5′ correction function, resulting in loss of replication fidelity and a high mutation frequency that leads to genome instability [82].

According to published data, EC’s *high-microsatellite-instability genetic subtype*, also known as *dMMR/MSI-H* EC, accounts for 30% of cases and results from a mutation in mutator genes (*dMMR*). They are observed in an age-heterogenic group of women with normal BMI and have an intermediate prognosis, and lymphatic vascular space infiltration is often observed. Microsatellite sequences are short-tandem repeats in both coding and non-coding regions of the genome and play a promoter role in the DNA replication process. Accumulations of errors in them lead to malfunction of the post-replication DNA repair system and microsatellite instability (*MSI*) [83].

*The high-copy-number subtype* is connected with older age and lower BMI and constitutes 15% of all EC. It has a worse prognosis with around a 60% mortality rate. Genetically it is characterized by high somatic copy number changes and is associated with a mutation in the TP53 gene, which encodes the p53 protein. This protein is responsible for the genetic material’s stability, the correct gene transcription, and DNA repair. EC with p53 abn mutation is characterized by the synthesis of p53 protein with oncogenic functions, promoting proliferation and resistance to treatment [83,84].

*Low-copy-number EC* is the variant without *POLE*, *dMMR*, and *TP53* mutations and occurs in 50% of EC cases [81]. It is characterized by low somatic gene mutations, histologically with a low degree of malignancy and an intermediate prognosis. The patients are with the highest BMI, as well as a high expression of estrogen receptors (ERs) and progesterone receptors (PRs). The most common mutation in this subtype is *CTNNB1,* but other mutations are also observed, as in the *mTOR* signaling pathway, associated with ER+ and PR+ variants [85].

### circRNAs and EC

In the process of formation of an organism, large numbers of cells are generated, and they obtain functional and morphological characteristics at a proper place and a proper time. Thus tissues and organs are formed. The final stage is a differentiated cell in a post-mitotic state. These temporal successive processes of proliferation, cell cycle withdrawal and differentiation are important for normal development and tissue homeostasis throughout the whole life cycle. The failure to arrest proliferation and lack of differentiation mark cancer cells’ major characteristics. This uncontrollable proliferation is accepted to be the major mechanism for carcinogenesis. We focus on the molecular mechanisms, in particular circRNAs, and their potential impact on the development and progression of endometrial cancer [86].

**Figure 5:**
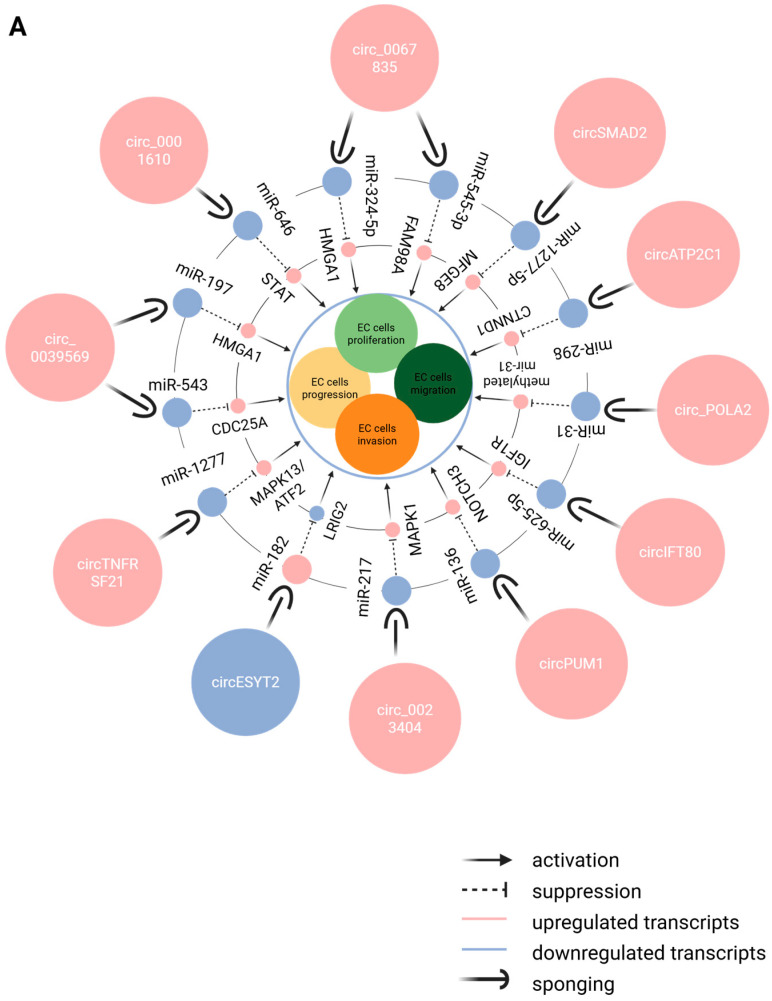

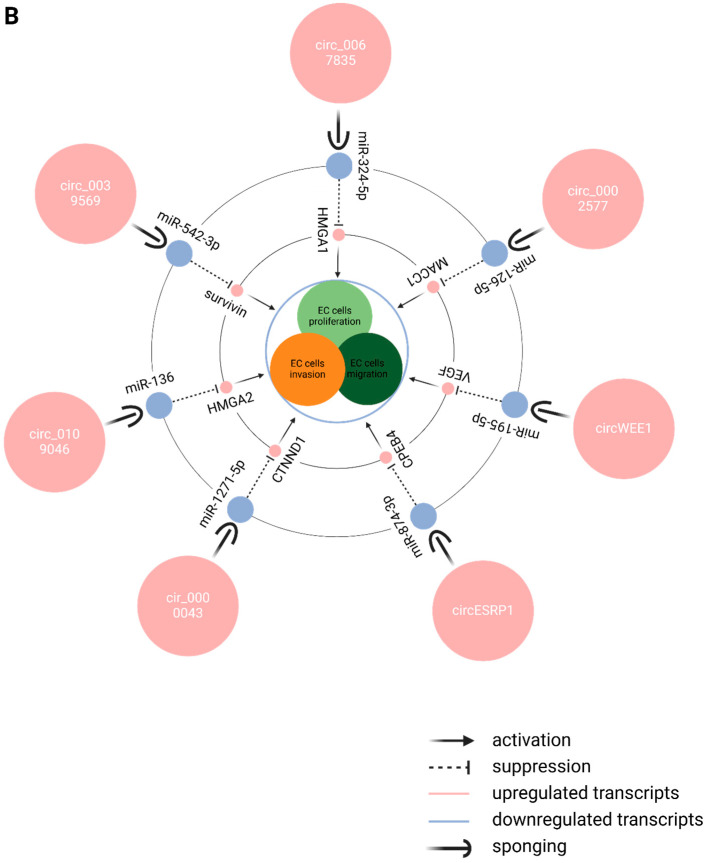

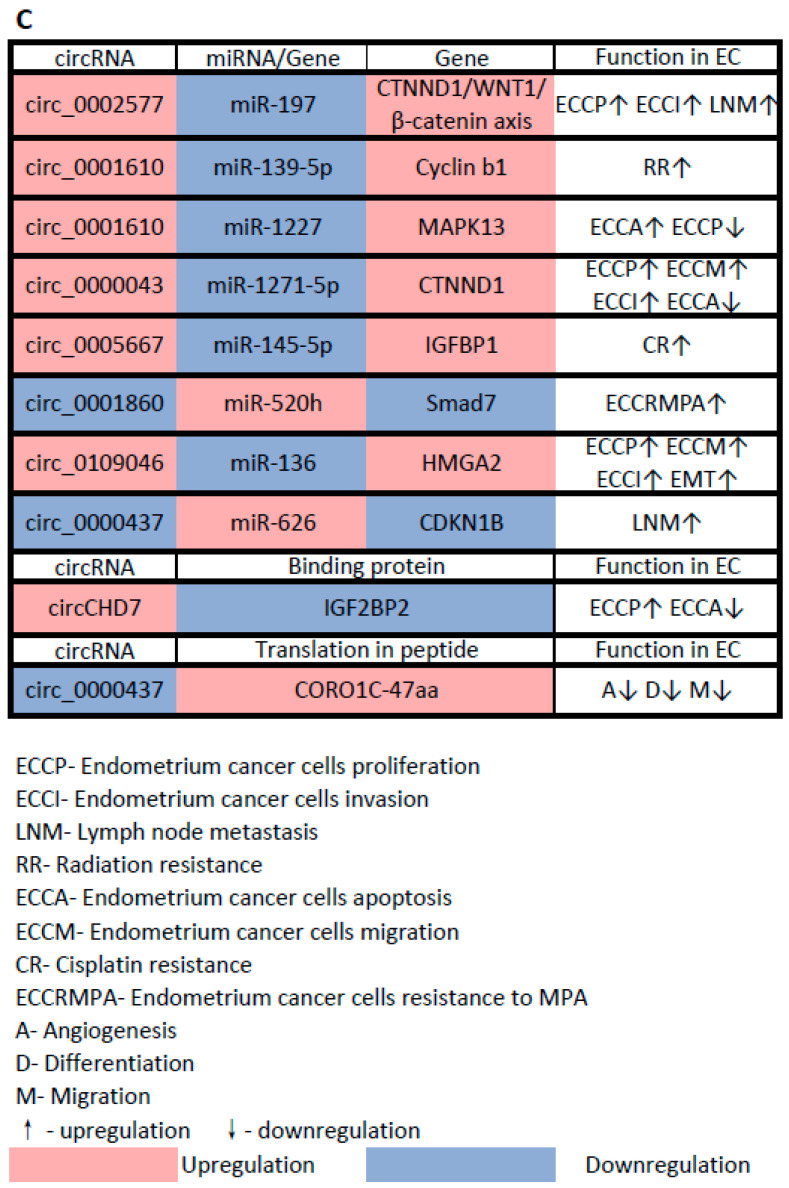
(**A**) CircRNA–miRNA–genes that influence four EC characteristics (EC cell proliferation, EC cell progression, EC cell invasion, and EC cell migration) in endometrial cancer (EC). (**B**) CircRNA–miRNA–genes that influence three EC characteristics (EC cell proliferation, EC cell invasion and EC cell migration) in endometrial cancer (EC) [90]. (**C**) CircRNA–miRNA–gene expressions that exert several functions in endometrial cancer (EC) [90]. Created in Biorender. Nikolova, M. (2025) https://BioRender.com/30my0db and https://BioRender.com/pvfwwjd).

There are few research studies concerning the role of circRNAs in EC. Their potential contribution to underlying molecular mechanisms in EC could be compared with other small non-coding RNAs like miRNAs and piRNAs (PIWI-interacting RNAs). It is interesting to compare their expression with other endometrial states—in health, transition to disease (like in endometrial hyperplasia) and disease. More and more data has been gathered with the help of high-throughput technologies, and the number of newly discovered circRNAs is increasing. Researchers have shown that over 70,000 cirRNAs have altered expression in EC tissues [86]. Ye et al. have found a correlation between hsa_circ_0039569 and two miRNAs. Hsa_circ_0039569 has MREs (miRNA response elements) for hsa-miR-542-3p and hsa-let-let-7c-5p and leads to their downregulation as well (Figure 5A). Significant downregulation of this circular RNA expression was detected in stage 3 of EC compared to stage 1. The correlation of some RNA-binding proteins with circRNAs was studied [87,88]. An interesting positive correlation was found between QKI’s (kH domain RNA binding protein) expression level and 35 circRNAs. QKI is known to induce EMT (epithelial-mesenchymal transition). MiRNA-binding sites have been predicted in the circRNAs. A set of known and predicted miRNAs subject to circRNA regulation were downregulated: miR200c, miR221, miR130a, miR130b, and 183. This correlation of circRNA-QKI-miRNAs, in which the upregulation of QKI leads to the upregulation of circRNAs and downregulation of certain miRNAs, is believed to be a possible molecular mechanism for EMT in EC [88]. Circ_PUM1 (circ_0000043) high expression promotes proliferation, metastasis, and invasion of EC cells; on the contrary, the knockdown of circ_PUM1 is followed by a reduction in tumor growth. It is known that circ_PUM1 regulates miR-136, which targets NOTCH3, a proven oncogene of EC; thus, the sponging of miR-136 by the circular RNA leads to the activation of NOTCH3 and activates the development of EC (Figure 5B) [89].

The Wnt/β-Catenin pathway has been proven as a signaling pathway connected with circ_0002577, which is thought to be involved in the proliferation and migration of EC cells [90]. It has been shown in a study that hsa_circ_001860 sponges miR-520h and thus leads to higher expression of Smad7, which is a suspected target of EC resistance to MPA (Medroxyprogesterone acetate). Resistance to MPA is negatively correlated with EC stage progress and lymph node metastasis [91]. Evidence has been gathered that the low expression of circ_0005667 makes EC cells more sensitive to cisplatin and leads to decreased proliferation, migration, and invasion of cancer cells [92] (Figure 5C).

CircRNAs have the potential to be used as biomarkers from liquid biopsies, as in blood or urine, but further studies about the correlation between circRNAs and EC are necessary [93]. This small RNA species has diagnostic, therapeutic, and prognostic potential for EC investigation, progression, and prognosis.

## 7. Discussion

CircRNAs are small regulatory molecules that participate in the epigenetic regulation of several processes in the organism, both in physiology and pathology. Their small size, stability, and impact on biological processes are characteristics that make them identifiable and reliable potential biomarkers and therapeutic targets. They are tissue-specific and pathologically changed-tissue-specific, cannot be degraded by endonucleases, and are stable in formalin-fixed paraffin-embedded tissue, which widens the opportunities for investigation and comparison of results. Thanks to the efficiency of high-throughput technologies and bioinformatics advances, many circRNAs are being identified. Yet there is a lack of a panoramic view of the circRNAs in human endometrium in the different phases of the endometrial cycle and pathologically changed endometrium. Some signaling pathways have been known to be significant for processes in the endometrium, allowing the realization of fine molecular mechanisms.

***The Wnt/β-Catenin pathway:*** The implantation of the embryo depends on the adhesion of trophoblast cells to the epithelial layer of the endometrium, as well as the cell-to-cell adhesion molecule interactions, in which the expression of β-catenin and some cadherins was found to be significantly lower in patients with RIF compared to the receptive endometrium of fertile patients [94]. Wnt/β-catenin signaling activation, occurring in the luminal epithelium at the potential site of implantation, requires the presence of the blastocyst. Furthermore, inhibition of Wnt/β-catenin signaling interferes with implantation, and the Wnt/β-catenin signaling pathway plays a central role in coordinating utero–embryo interactions necessary for implantation [95]. β-catenin and MYC1 have been identified as targets of miR-let-7-a/g in mice [96]. MiR-let-7 is a microRNA that is considered highly conserved across species. Although the two microRNA families miR-let-7-a and miR-let-7-g can influence mouse endometrial receptivity, only miR-let-7-a is associated with human endometrial receptivity [97]. The Wnt signaling pathway impaired endometrial receptivity due to miR-let-7-a/g’s negative impact on β-catenin synthesis, and the suppression of the Wnt signaling pathway resulted in improved endometrial receptivity in mice [97]. MiR-200c-3p was found to affect endometrial receptivity by indirectly targeting the Wnt signaling pathway [98]. Circ_0007331 was identified as a miRNA sponge for miR-200c-3p to indirectly regulate the function of HIF-1α, which plays a central role in the local angiogenesis and hypoxic mechanisms in the endometrium. A knock-down of circ_0007331 could suppress the development of endometrium through down-regulation of the expression of HIF-1α. The axis circ_0007331/miR-200c-3p/HIF-1α could affect the proliferation and invasion of endometrial cells [99]. The Wnt/β-Catenin pathway has been proven as a signaling pathway connected with the circ_0002577/miR-197/CTNND1 axis, which is related to the proliferation and migration of EC cells [90].

***NOTCH and Wnt signaling pathways:*** In the context of endometrial remodeling, there is accumulating evidence of interactions between the NOTCH and Wnt signaling pathways. The NOTCH signaling pathway has impacts on the determination of cell fate. In a clinical study, compared with healthy fertile women, patients with endometriosis, RIF, and polycystic ovary syndrome (PCOS) showed dysregulated NOTCH signaling expression in the mid-luteal phase [100]. Other evidence in agreement with that from the clinical study shows that the NOTCH and Wnt signaling pathways influence the proportion of ciliated and secretory cells. Maps of the temporal dynamics of the human endometrium have been created [101]. Based on these, the opposite roles of Wnt and NOTCH for the specific fate of differentiating cells are proven. Wnt dominates during the early secretory phase, thereby maintaining the ciliary epithelial lineage. In contrast, NOTCH dominates in the middle and late secretory phases to drive the differentiation of secretory epithelial cells.

It is interesting to mention miR-449, which is part of the miR-34/449 superfamily, which includes six homologous miRs (miR-34a, miR-34b/c, and miR-449a/b/c). The isoforms associated with a receptive endometrium are 5′-isoforms of miR-449c, miR-34a/c, and miR-449a/b. MiR-449a/b has a far more pronounced effect on cell proliferation and migration in cancer compared to miR-449c [102]. Since the 5′-isoforms of miR-449c associated with endometrial receptivity share the same target genes with miR-34a/c and miR-449a/b, we believe that these isoforms may be involved in the control of different proliferative activities of the cells in the endometrium. Among the differentially expressed genes in the receptive endometrium, MYCN (v-myc myelocytomatosis viral-related oncogene, neuroblastoma-derived (avian)) was predicted as a target gene of the 5′-isoforms of miR-449c and let7g-5p. A regulatory network involving miR-449c and MYCN can be hypothesized in the receptive endometrium [103].

A negative relationship was found between the expression levels of MYCN (decreased) and the isoforms of these microRNAs (increased) in the receptive phase, suggesting a functional relevance of the miR-449c isoform family as a negative regulator of MYCN. This gene is a member of the MYC (Myelocytomatosis oncogene) family, and its function is to increase or decrease gene expression directly by binding to the promoter or indirectly through mediators [104]. There is now much evidence that MYC proteins play a role as transcription factors. The study of MYC-dependent RNA levels indicates that MYC proteins enhance the expression or repress specific target genes [105]. MYC genes also include the MYCN gene (N-myc proto-oncogene), which is also a transcription modulator and is found significantly more often in estrogen-dependent endometrial carcinoma than MYC. The higher its expression is, the poorer the prognosis for the development of endometrial carcinoma [105].

**Figure 6:**
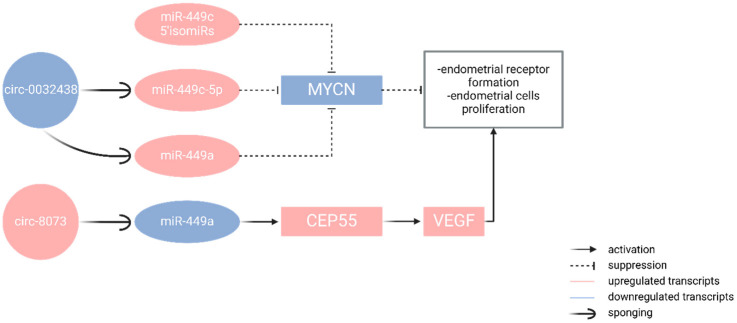
Interactions of ciRNAs and miRNAs with target transcripts in receptive and cancerous endometrium. The low expression profile of circ_0032438 leads to high expression profiles of miR-449c-5p and miR-449a, which, together with miR-449c 5′ isoform, downregulate the expression of MYCN, which results in suppression of endometrial receptor formation and EC cell proliferation. The upregulated circ_8073 sponges miR-449a, which leads to its low expression and the activation of CEP55 and VEGF, which induce endometrial receptor formation and EC cell proliferation. (Created in Biorender. Nikolova, M. (2025) https://BioRender.com/901j0es).

Some studies showed that circ-8073 directly binds miR-449a and inhibits its activity in caprine endometrial cells in vitro [106]. Centrosomal protein 55 (CEP55) was found to be a direct target of miR-449a. Circ-8073 improved the expression of CEP55, sponging miR-449a in endometrial epithelial cells in vitro. And the axis circ-8073/miR-449a/CEP55 promotes endometrial cancer proliferation via the PI3K/AKT/mTOR pathway. Additionally, CEP55 regulates the expression of VEGF and FOXM1 in endometrial epithelial cells and thus contributes to the formation of endometrial receptors. These findings suggest that circ-8073 regulates endometrial receptivity and endometrial cancer proliferation via miR-449a/CEP55 and PI3K/AKT/mTOR pathways. It has been speculated that circ-0032438 regulates both miR-449a and miR-449c-5p and represses proliferation and inflammation in the endometrium, and miR-449a is thought to activate local autophagy (Figure 6) [107,108].

There is evidence that some circRNAs, e.g., circ_0067301, circ_103470, circ_101102, and circATRNL1, can promote EMT in endometriosis by sponging a common miRNA, miR-141. The inhibitory effects of miR-141 on proliferation and/or migration and EMT have been proven in endometrial cells. Additionally, circ_0067301 and circ_0061140 can induce the expression of different members of the Notch family, Notch1 and Notch2, in endometriosis. Future studies could further investigate the hyperactivated action of the Notch family and the role of the circRNA-miRNA-Notch axis in EMT [109]. Circ_PUM1 high expression promotes proliferation, metastasis, and invasion of EC cells, and the knockdown of circ_PUM1 reduces tumor growth. Circ_PUM1 regulates miR-136, which targets NOTCH3, a proven oncogene of EC. Sponging of miR-136 by the circular RNA leads to the activation of NOTCH3 and activates the development of EC [89].

Lack of Circ_0008433 represses proliferation, migration, and angiogenesis and promotes apoptosis in endometrial stromal cells [110]. MiRNAs, potential targets of this circular RNA, e.g., miR-221-3p, miR-222-3p, miR-181-5p, miR-449a, miR-449b-5p, and miR-483-3p, were significantly changed with overexpression of circ_0008433 in endometrial stromal cells. The up-regulation of circ_0008433 can modulate EMT through the circRNA–miRNA–mRNA axis [110].

**In vitro *attempts to target circRNAs***

Several experimental strategies have been considered to target circular RNAs for therapeutic reasons. These strategies aim to achieve overexpression or knockdown/knockout.

**Figure 7:**
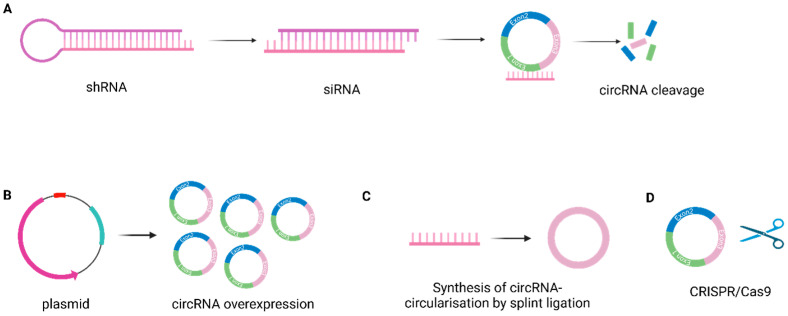
In vitro *therapeutic* targeting strategies for circular RNA overexpression or knockdown/knockout (**A**). Short hairpin RNAs bind complimentary to cirRNAs and cleave them, (**B**). CircRNAs overexpression achievement through plasmids, (**C**). Direct synthesis of cirRNA from linear RNA, (**D**). CRISPR/Cas 9 mediated knockout or knockdown (created in Biorender. Nikolova, M. (2025) https://BioRender.com/ys4yyfa).

One potential strategy is *RNA-interference-mediated circular RNA knockdown*. Double-stranded short interfering RNAs (siRNAs) or short hairpin RNAs (shRNAs) bind complimentary to circRNAs, include them in the RNA-induced silencing complex, and cleave them. Usually, the back-splicing junction is targeted to ensure specificity and not affect the linear cognates [111] (Figure 7A). For the purposes of the achievement of *overexpression* of circRNAs, plasmids have been used (Figure 7B). In the *synthesis of circRNAs*, single-stranded linear RNA molecules can be synthesized and then circularized by splint ligation (Figure 7C) [112]. *CRISPR/Cas 9-mediated knockout or knockdown* (Clustered Regularly Interspaced Short Palindromic Repeats) is another strategy characterized by high specificity and efficiency. It uses small guide RNAs (gRNA), which direct Cas9 nuclease to pair and disrupt the introns that flank the circularizing exons in biogenesis. Due to specificity, the linear transcripts are not affected [113] (Figure 7D).

All these therapeutic approaches have been applied in preclinical studies, either in vitro or on animal models. There are many obstacles yet, and their clinical application is still a potential goal. ***Nanoparticles*** can be used to carry and deliver small molecules to targeted tissue as therapeutic agents, and these carriers can be engineered in size and characteristics. They can be liposomes, polymers, or dendrimers [114].

***Exosomes*** can be used as vehicles for the delivery of circRNAs-targeting agents. Exosomes are small extracellular vesicles of 30–100 nm diameter, produced and accepted by cells, and thus intercellular communication is realized [115,116]. There is an interesting observation that cancer cells secrete much more exosomes than healthy cells [116]. Exosomes can carry different molecules, including circRNAs, miRNAs, long non-coding RNAs, proteins, and lipids [117]. Both exosomes and nanoparticles can deliver the inclusive molecules, protecting them from degradation and allowing them to reach the targeted cells without triggering the immune system.

## 8. Conclusions

The increasing data gathered thanks to high-throughput technologies and bioinformatic analysis will allow the construction of regulatory circRNA–miRNA–mRNA networks, as well as assessment of their impact on both receptive and cancerous endometria. That new knowledge can be transferred from preclinical studies to clinical applications. The currently available data have some limitations due to the small sample size and the lack of clinical validations. Studies based on interspecies conservatism of genes should be interpreted with caution. Predominantly, these studies are still in a preclinical phase. The molecular analysis of endometrial biopsy material is associated with invasive procedures, and new low or noninvasive routes of material achievement are being searched. Collections of uterine fluid or blood are considered low-invasive procedures. There are some challenges because of the low analyte concentration, which can make the extraction and purification of RNA difficult, and some enrichment strategies need to be applied. EC’s heterogeneity makes the interpretation of the results more difficult. Yet there are still no standards for uterine liquid recruitment, processing, and analysis, and this could be a future technical goal, as it can significantly influence the results.

The accumulation of more data on circRNAs and a deeper analysis could show a new level of epigenetic regulation and a better understanding of both receptive and cancerous endometrium. CircRNAs have the potential to be biomarkers and therapeutic targets for difficult RIF cases, and their solution can increase the chances for pregnancy achievement in ART. They can find a place in clinical practice as both new diagnostic and therapeutic tools for EC diagnosis, therapy, progression follow-up, and prognosis.

## Figures and Tables

**Table 1:** KEGG pathway analysis of six DEmRNAs and modifications (*p*-value ranged between 1.58 and 6.83 and enrichment ranged between 56.80 and 341.04) [68].

KEGG Pathway	HitGenes
HIF-1 signaling pathway	HIF1A, IGF1R, PIK3R1, MAP2K1, VEGFA
Focal adhesion	CDC42, IGF1R, PIK3R1, MAP2K1, VEGFA
VEGF signaling pathway	CDC42, PIK3R1, MAP2K1, VEGFA
Autophagy—animal	HIF1A, IGF1R, PIK3R1, MAP2K1
PI3K-Akt signaling pathway	IGF1R, PIK3R1, MAP2K1, VEGFA
mTOR signaling pathway	IGF1R, PIK3R1, MAP2K1

## Data Availability

Publicly available data has been analyzed in this study.

## References

[1] Chen L.-L., Yang L. (2015). Regulation of circRNA biogenesis. RNA Biol..

[2] Enuka Y., Lauriola M., Feldman M.E., Sas-Chen A., Ulitsky I., Yarden Y. (2016). Circular RNAs are long-lived and display only minimal early alterations in response to a growth factor. Nucleic Acids Res..

[3] Guo J.U., Agarwal V., Guo H., Bartel D.P. (2014). Expanded identification and characterization of mammalian circular RNAs. Genome Biol..

[4] Glažar P., Papavasileiou P., Rajewsky N. (2014). circBase: A database for circular RNAs. RNA.

[5] Ghosal S., Das S., Sen R., Basak P., Chakrabarti J. (2013). Circ2Traits: A comprehensive database for circular RNA potentially associated with disease and traits. Front. Genet..

[6] Zhao R.-T., Zhou J., Dong X.-L., Bi C.-W., Jiang R.-C., Dong J.-F., Tian Y., Yuan H.-J., Zhang J.-N. (2018). Circular ribonucleic acid expression alteration in exosomes from the brain extracellular space after traumatic brain injury in mice. J. Neurotrauma.

[7] Ivanov A., Memczak S., Wyler E., Torti F., Porath H.T., Orejuela M.R., Piechotta M., Levanon E.Y., Landthaler M., Dieterich C. (2015). Analysis of intron sequences reveals hallmarks of circular RNA biogenesis in animals. Cell Rep..

[8] Ashwal-Fluss R., Meyer M., Pamudurti N.R., Ivanov A., Bartok O., Hanan M., Evantal N., Memczak S., Rajewsky N., Kadener S. (2014). circRNA biogenesis competes with pre-mRNA splicing. Mol. Cell.

[9] Li Z., Huang C., Bao C., Chen L., Lin M., Wang X., Zhong G., Yu B., Hu W., Dai L. (2015). Exon-intron circular RNAs regulate transcription in the nucleus. Nat. Struct. Mol. Biol..

[10] Capel B., Swain A., Nicolis S., Hacker A., Walter M., Koopman P., Goodfellow P., Lovell-Badge R. (1993). Circular transcripts of the testis-determining gene Sry in adult mouse testis. Cell.

[11] Kramer M.C., Liang D., Tatomer D.C., Gold B., March Z.M., Cherry S., Wilusz J.E. (2015). Combinatorial control of *Drosophila* circular RNA expression by intronic repeats, hnRNPs, and SR proteins. Genes Dev..

[12] Jeck W.R., Sorrentino J.A., Wang K., Slevin M.K., Burd C.E., Liu J., Marzluff W.F., Sharpless N.E. (2013). Circular RNAs are abundant, conserved, and associated with ALU repeats. RNA.

[13] Zhang X.O., Wang H.B., Zhang Y., Lu X., Chen L.L., Yang L. (2014). Complementary sequence-mediated exon circularization. Cell.

[14] Zhang Y., Xue W., Li X., Zhang J., Chen S., Zhang J.-L., Yang L., Chen L.-L. (2016). The biogenesis of nascent circular RNAs. Cell Rep..

[15] Conn S.J., Pillman K.A., Toubia J., Conn V.M., Salmanidis M., Phillips C.A., Roslan S., Schreiber A.W., Gregory P.A., Goodall G.J. (2015). The RNA binding protein quaking regulates formation of circRNAs. Cell.

[16] Liu D., Dredge B.K., Bert A.G., Pillman K.A., Toubia J., Guo W., Dyakov B.J.A., Migault M.M., Conn V.M., Conn S.J. (2024). ESRP1 controls biogenesis and function of a large abundant multiexon circRNA. Nucleic Acids Res..

[17] Bonczek O., Wang L., Gnanasundram S.V., Chen S., Haronikova L., Zavadil-Kokas F., Vojtesek B. (2022). DNA and RNA binding proteins: From motifs to roles in cancer. Int. J. Mol. Sci..

[18] Stoll L., Sobel J., Rodriguez-Trejo A., Guay C., Lee K., Venø M.T., Kjems J., Laybutt D.R., Regazzi R. (2018). Circular RNAs as novel regulators of β-cell functions in normal and disease conditions. Mol. Metab..

[19] Geng H.-H., Li R., Su Y.-M., Xiao J., Pan M., Cai X.-X., Ji X.-P. (2016). The circular RNA Cdr1as promotes myocardial infarction by mediating the regulation of miR-7a on its target genes expression. PLoS ONE.

[20] Zhang J., Hu H., Zhao Y., Zhao Y. (2018). CDR1as is overexpressed in laryngeal squamous cell carcinoma to promote the tumour’s progression via miR-7 signals. Cell Prolif..

[21] Han S., Zhang T., Kusumanchi P., Huda N., Jiang Y., Liangpunsakul S., Yang Z. (2020). Role of microRNA-7 in liver diseases: A comprehensive review of the mechanisms and therapeutic applications. J. Investig. Med..

[22] Huang G., Zhu H., Shi Y., Wu W., Cai H., Chen X. (2015). cir-ITCH plays an inhibitory role in colorectal cancer by regulating the Wnt/β-catenin pathway. PLoS ONE.

[23] Wang M., Chen B., Ru Z., Cong L. (2018). CircRNA circ-ITCH suppresses papillary thyroid cancer progression through miR-22-3p/CBL/β-catenin pathway. Biochem. Biophys. Res. Commun..

[24] Du W.W., Yang W., Chen Y., Wu Z.-K., Foster F.S., Yang Z., Li X., Yang B.B. (2017). Foxo3 circular RNA promotes cardiac senescence by modulating multiple factors associated with stress and senescence responses. Eur. Heart J..

[25] Du W.W., Yang W., Li X., Awan F.M., Yang Z., Fang L., Lyu J., Li F., Peng C., Krylov S.N. (2018). A circular RNA circ-DNMT1 enhances breast cancer progression by activating autophagy. Oncogene.

[26] Wang Y., Wang Z. (2015). Efficient backsplicing produces translatable circular mRNAs. RNA.

[27] Pamudurti N.R., Bartok O., Jens M., Ashwal-Fluss R., Stottmeister C., Ruhe L., Hanan M., Wyler E., Perez-Hernandez D., Ramberger E. (2017). Translation of circRNAs. Mol. Cell.

[28] Legnini I., Di Timoteo G., Rossi F., Morlando M., Briganti F., Sthandier O., Fatica A., Santini T., Andronache A., Wade M. (2017). Circ-ZNF609 is a circular RNA that can be translated and functions in myogenesis. Mol. Cell.

[29] Zhang M., Zhao K., Xu X., Yang Y., Yan S., Wei P., Liu H., Xu J., Xiao F., Zhou H. (2018). A peptide encoded by circular form of LINC-PINT suppresses oncogenic transcriptional elongation in glioblastoma. Nat. Commun..

[30] Homem C.C.F., Repic M., Knoblich J.A. (2015). Proliferation control in neural stem and progenitor cells. Nat. Rev. Neurosci..

[31] Shan K., Liu C., Liu B.-H., Chen X., Dong R., Liu X., Zhang Y.-Y., Liu B., Zhang S.-J., Wang J.-J. (2017). Circular noncoding RNA HIPK3 mediates retinal vascular dysfunction in diabetes mellitus. Circulation.

[32] Shen Y., Zhao N., Hu X., He X., Xu Y., Chen J., Chen W., Liu X., Zhou Z., Cao D. (2022). Tumor-suppressive and oncogenic roles of microRNA-149-5p in human cancers. Int. J. Mol. Sci..

[33] Zheng Q., Bao C., Guo W., Li S., Chen J., Chen B., Luo Y., Lyu D., Li Y., Shi G. (2016). Circular RNA profiling reveals an abundant circHIPK3 that regulates cell growth by sponging multiple miRNAs. Nat. Commun..

[34] Zhong Z., Lv M., Chen J. (2016). Screening differential circular RNA expression profiles reveals the regulatory role of circTCF25-miR-103a-3p/miR-107-CDK6 pathway in bladder carcinoma. Sci. Rep..

[35] Zhang Z., Yang T., Xiao J. (2018). Circular RNAs: Promising biomarkers for human diseases. EBioMedicine.

[36] Wu Z., Shi W., Jiang C. (2018). Overexpressing circular RNA hsa_circ_0002052 impairs osteosarcoma progression via inhibiting Wnt/β-catenin pathway by regulating miR-1205/APC2 axis. Biochem. Biophys. Res. Commun..

[37] Lamouille S., Xu J., Derynck R. (2014). Molecular mechanisms of epithelial–mesenchymal transition. Nat. Rev. Mol. Cell Biol..

[38] Dai Y., Li D., Chen X., Tan X., Gu J., Chen M., Zhang X. (2018). Circular RNA myosin light chain kinase (MYLK) promotes prostate cancer progression through modulating Mir-29a expression. Med. Sci. Monit..

[39] Zhang X., Luo P., Jing W., Zhou H., Liang C., Tu J. (2018). circSMAD2 inhibits the epithelial–mesenchymal transition by targeting miR-629 in hepatocellular carcinoma. OncoTargets Ther..

[40] Yu C.-Y., Li T.-C., Wu Y.-Y., Yeh C.-H., Chiang W., Chuang C.-Y., Kuo H.-C. (2017). The circular RNA circBIRC6 participates in the molecular circuitry controlling human pluripotency. Nat. Commun..

[41] Memczak S., Jens M., Elefsinioti A., Torti F., Krueger J., Rybak A., Maier L., Mackowiak S.D., Gregersen L.H., Munschauer M. (2013). Circular RNAs are a large class of animal RNAs with regulatory potency. Nature.

[42] Li H., Wei X., Yang J., Dong D., Hao D., Huang Y., Lan X., Plath M., Lei C., Ma Y. (2018). circFGFR4 promotes differentiation of myoblasts via binding miR-107 to relieve its inhibition of Wnt3a. Mol. Ther.-Nucleic Acids.

[43] Li X., Zheng Y., Zheng Y., Huang Y., Zhang Y., Jia L., Li W. (2018). Circular RNA CDR1as regulates osteoblastic differentiation of periodontal ligament stem cells via the miR-7/GDF5/SMAD and p38 MAPK signaling pathway. Stem Cell Res. Ther..

[44] https://histology.siu.edu.

[45] Gray C.A., Bartol F.F., Tarleton B.J., Wiley A.A., Johnson G.A., Bazer F.W., Spencer T.E. (2001). Developmental biology of uterine glands. Biol. Reprod..

[46] Fu X.-D. (2014). Non-coding RNA: A new frontier in regulatory biology. Natl. Sci. Rev..

[47] Garcia-Alonso L., Handfield L.-F., Roberts K., Nikolakopoulou K., Fernando R.C., Gardner L., Woodhams B., Arutyunyan A., Polanski K., Hoo R. (2021). Mapping the temporal and spatial dynamics of the human endometrium in vivo and in vitro. Nat. Genet..

[48] Nusse R., Varmus H. (2012). Three decades of Wnts: A personal perspective on how a scientific field developed. EMBO J..

[49] Komiya Y., Habas R. (2008). Wnt signal transduction pathways. Organogenesis.

[50] Dexter J.S. (1914). The analysis of a case of continuous variation in Drosophila by a study of its linkage relations. Am. Nat..

[51] Kumar R., Juillerat-Jeanneret L., Golshayan D. (2016). Notch antagonists: Potential modulators of cancer and inflammatory diseases. J. Med. Chem..

[52] Lowell S., Jones P., Le Roux I., Dunne J., Watt F.M. (2000). Stimulation of human epidermal differentiation by Delta–Notch signalling at the boundaries of stem-cell clusters. Curr. Biol..

[53] Hellström M., Phng L.-K., Hofmann J.J., Wallgard E., Coultas L., Lindblom P., Alva J., Nilsson A.-K., Karlsson L., Gaiano N. (2007). Dll4 signalling through Notch1 regulates formation of tip cells during angiogenesis. Nature.

[54] Bolós V., Grego-Bessa J., De La Pompa J.L. (2007). Notch signaling in development and cancer. Endocr. Rev..

[55] Orchard M.D., Murphy C.R. (2002). Alterations in tight junction molecules of uterine epithelial cells during early pregnancy in the rat. Acta Histochem..

[56] Murphy C.R., Hosie M.J., Thompson M.B. (2000). The plasma membrane transformation facilitates pregnancy in both reptiles and mammals. Comp. Biochem. Physiol. Part A Mol. Integr. Physiol..

[57] Whitby S., Zhou W., Dimitriadis E. (2020). Alterations in epithelial cell polarity during endometrial receptivity: A systematic review. Front. Endocrinol..

[58] Cha J., Sun X., Dey S.K. (2012). Mechanisms of implantation: Strategies for successful pregnancy. Nat. Med..

[59] Ruiz-Alonso M., Blesa D., Simón C. (2012). The genomics of the human endometrium. Biochim. Biophys. Acta (BBA)-Mol. Basis Dis..

[60] Sebastian-Leon P., Garrido N., Remohí J., Pellicer A., Diaz-Gimeno P. (2018). Asynchronous and pathological windows of implantation: Two causes of recurrent implantation failure†. Hum. Reprod..

[61] Simon A., Laufer N. (2012). Assessment and treatment of repeated implantation failure (RIF). J. Assist. Reprod. Genet..

[62] Altmäe S., Martinez-Conejero J.A., Esteban F.J., Ruiz-Alonso M., Stavreus-Evers A., Horcajadas J.A., Salumets A. (2013). MicroRNAs miR-30b, miR-30d, and miR-494 regulate human endometrial receptivity. Reprod. Sci..

[63] Kim A., Jung H., Choi W.J., Hong S.N., Kim H.Y. (2014). Detection of endometrial and subendometrial vasculature on the day of embryo transfer and prediction of pregnancy during fresh in vitro fertilization cycles. Taiwan. J. Obstet. Gynecol..

[64] Kasius A., Smit J.G., Torrance H.L., Eijkemans M.J., Mol B.W., Opmeer B.C., Broekmans F.J. (2014). Endometrial thickness and pregnancy rates after IVF: A systematic review and meta-analysis. Hum. Reprod. Updat..

[65] Yu C.-Y., Kuo H.-C. (2019). The emerging roles and functions of circular RNAs and their generation. J. Biomed. Sci..

[66] Liu L., Li L., Ma X., Yue F., Wang Y., Wang L., Jin P., Zhang X. (2018). Altered circular RNA expression in patients with repeated implantation failure. Cell. Physiol. Biochem..

[67] Wang A., Chen P. (2024). Comprehensive analysis of circRNA-miRNA-mRNA network related to angiogenesis in recurrent implantation failure. BMC Med. Genom..

[68] Liu X., Zhang L., Liu Y., Cui J., Che S., An X., Song Y., Cao B. (2018). Circ-8073 regulates CEP55 by sponging miR-449a to promote caprine endometrial epithelial cells proliferation via the PI3K/AKT/mTOR pathway. Biochim. Biophys. Acta (BBA)-Mol. Cell Res..

[69] Lin S.-C., Wang C.-C., Wu M.-H., Yang S.-H., Li Y.-H., Tsai S.-J. (2012). Hypoxia-induced microRNA-20a expression increases ERK phosphorylation and angiogenic gene expression in endometriotic stromal cells. J. Clin. Endocrinol. Metab..

[70] Salmasi S., Sharifi M., Rashidi B. (2021). Ovarian stimulation and exogenous progesterone affect the endometrial miR-16-5p, VEGF protein expression, and angiogenesis. Microvasc. Res..

[71] Mladinov D., Liu Y., Mattson D.L., Liang M. (2013). MicroRNAs contribute to the maintenance of cell-type-specific physiological characteristics: miR-192 targets Na^+^/K^+^-ATPase β1. Nucleic Acids Res..

[72] He X., Liu N., Mu T., Lu D., Jia C., Wang S., Yin C., Liu L., Zhou L., Huang X. (2020). Oestrogen induces epithelial-mesenchymal transition in endometriosis via circ_0004712/miR-148a-3p sponge function. J. Cell. Mol. Med..

[73] Burke W.M., Orr J., Leitao M., Salom E., Gehrig P., Olawaiye A.B., Brewer M., Boruta D., Herzog T.J., Abu Shahin F. (2014). Endometrial cancer: A review and current management strategies: Part II. Gynecol. Oncol..

[74] Jones E.R., O’Flynn H., Njoku K., Crosbie E.J. (2021). Detecting endometrial cancer. Obstet. Gynaecol..

[75] Lu K.H., Broaddus R.R. (2020). Endometrial cancer. N. Engl. J. Med..

[76] Gao J., Fan Y.-Z., Gao S.-S., Zhang W.-T. (2023). Circulating microRNAs as Potential Biomarkers for the Diagnosis of Endometrial Cancer: A Meta-Analysis. Reprod. Sci..

[77] McCluggage W.G., Bosse T., Gilks C.B., Howitt B.E., McAlpine J.N., Nucci M.R., Rabban J.T., Singh N., Talia K.L., Parra-Herran C. (2024). FIGO 2023 endometrial cancer staging: Too much, too soon?. Int. J. Gynecol. Cancer.

[78] Vergote I., Matias-Guiu X. (2023). New FIGO 2023 endometrial cancer staging validation. Welcome to the first molecular classifiers and new pathological variables!. Eur. J. Cancer.

[79] Weinstein J.N., Collisson E.A., Mills G.B., Shaw K.R.M., Ozenberger B.A., Ellrott K., Shmulevich I., Sander C., Stuart J.M., The Cancer Genome Atlas Research Network (2013). The cancer genome atlas pan-cancer analysis project. Nat. Genet..

[80] Yang Y., Wu S.F., Bao W. (2024). Molecular subtypes of endometrial cancer: Implications for adjuvant treatment strategies. Int. J. Gynecol. Obstet..

[81] Selves J., e Gloria H.D.C., Brunac A.-C., Saffi J., Guimbaud R., Brousset P., Hoffmann J.-S. (2024). Exploring the basis of heterogeneity of cancer aggressiveness among the mutated POLE variants. Life Sci. Alliance.

[82] Kanopiene D., Vidugiriene J., Valuckas K.P., Smailyte G., Uleckiene S., Bacher J. (2014). Endometrial cancer and microsatellite instability status. Open Med..

[83] Jamieson A., Thompson E., Huvila J., Leung S., Lum A., Helpman L., Salvador S., Irving J., Grondin K., Lytwyn A. (2021). OP008/# 194 P53ABN molecular subtype encompasses a morphologically diverse subset of endometrial cancers and identifies therapeutic opportunities to improve outcomes. Int. J. Gynecol. Cancer.

[84] Ribeiro-Santos P., Vieira C.M., Veloso G.G.V., Giannecchini G.V., Arenhardt M.P., Gomes L.M., Zanuncio P., Brandão F.S., Nogueira-Rodrigues A. (2024). Tailoring endometrial cancer treatment based on molecular pathology: Current status and possible impacts on systemic and local treatment. Int. J. Mol. Sci..

[85] Ye F., Tang Q.L., Ma F., Cai L., Chen M., Ran X.X., Wang X.Y., Jiang X.F. (2019). Analysis of the circular RNA transcriptome in the grade 3 endometrial cancer. Cancer Manag. Res..

[86] Ruijtenberg S., van den Heuvel S. (2016). Coordinating cell proliferation and differentiation: Antagonism between cell cycle regulators and cell type-specific gene expression. Cell Cycle.

[87] Dou Y., Kawaler E.A., Zhou D.C., Gritsenko M.A., Huang C., Blumenberg L., Karpova A., Petyuk V.A., Savage S.R., Satpathy S. (2020). Proteogenomic characterization of endometrial carcinoma. Cell.

[88] Zong Z., Liu Y., Chen S., Zhao Y. (2020). Circ_PUM1 promotes the development of endometrial cancer by targeting the miR-136/NOTCH3 pathway. J. Cell. Mol. Med..

[89] Shen Q., He T., Yuan H. (2019). Hsa_circ_0002577 promotes endometrial carcinoma progression via regulating miR-197/CTNND1 axis and activating Wnt/β-catenin pathway. Cell Cycle.

[90] Włodarczyk K., Kuryło W., Pawłowska-Łachut A., Skiba W., Suszczyk D., Pieniądz P., Majewska M., Boniewska-Bernacka E., Wertel I. (2024). circRNAs in Endometrial Cancer—A Promising Biomarker: State of the Art. Int. J. Mol. Sci..

[91] Yuan S., Zheng P., Sun X., Zeng J., Cao W., Gao W., Wang Y., Wang L. (2021). Hsa_Circ_0001860 promotes Smad7 to enhance MPA resistance in endometrial cancer via miR-520h. Front. Cell Dev. Biol..

[92] Sun G., Tian J., Xiao Y., Zeng Y. (2023). Circular RNA circ_0005667 promotes cisplatin resistance of endometrial carcinoma cells by regulating IGF2BP1 through miR-145-5p. Anti-Cancer Drugs.

[93] Koler M., Achache H., Tsafrir A., Smith Y., Revel A., Reich R. (2009). Disrupted gene pattern in patients with repeated in vitro fertilization (IVF) failure. Hum. Reprod..

[94] Mohamed O.A., Jonnaert M., Labelle-Dumais C., Kuroda K., Clarke H.J., Dufort D. (2005). Uterine Wnt/β-catenin signaling is required for implantation. Proc. Natl. Acad. Sci. USA.

[95] Inyawilert W., Fu T.-Y., Lin C.-T., Tang P.-C. (2015). Let-7-mediated suppression of mucin 1 expression in the mouse uterus during embryo implantation. J. Reprod. Dev..

[96] Li Q., Liu W., Chiu P.C., Yeung W.S. (2020). Mir-let-7a/g enhances uterine receptivity via suppressing Wnt/β-catenin under the modulation of ovarian hormones. Reprod. Sci..

[97] Zheng Q., Zhang D., Yang Y.U., Cui X., Sun J., Liang C., Qin H., Yang X., Liu S., Yan Q. (2017). MicroRNA-200c impairs uterine receptivity formation by targeting FUT4 and α1,3-fucosylation. Cell Death Differ..

[98] Dong L., Zhang L., Liu H., Xie M., Gao J., Zhou X., Zhao Q., Zhang S., Yang J. (2020). Circ_0007331 knock-down suppresses the progression of endometriosis via miR-200c-3p/HiF-1alpha axis. J. Cell Mol. Med..

[99] Amjadi F.S., Salehi E., Zandieh Z., Rashidi M., Taleahmad S., Masrour M.J., Aflatoonian R., Mehdizadeh M. (2019). Comparative evaluation of NOTCH signaling molecules in the endometrium of women with various gynecological diseases during the window of implantation. Iran. J. Basic Med. Sci..

[100] Guo S., Quan S., Zou S. (2021). Roles of the notch signaling pathway in ovarian functioning. Reprod. Sci..

[101] Sandbothe M., Buurman R., Reich N., Greiwe L., Vajen B., Gürlevik E., Schäffer V., Eilers M., Kühnel F., Vaquero A. (2017). The microRNA-449 family inhibits TGF-β-mediated liver cancer cell migration by targeting SOX4. J. Hepatol..

[102] Nikolova M., Naydenov M., Glogovitis I., Apostolov A., Saare M., Boggavarapu N., Salumets A., Baev V., Yahubyan G. (2021). Coupling miR/isomiR and mRNA expression signatures unveils new molecular layers of endometrial receptivity. Life.

[103] Schwab M. (2004). MYCN in neuronal tumours. Cancer Lett..

[104] Baluapuri A., Wolf E., Eilers M. (2020). Target gene-independent functions of MYC oncoproteins. Nat. Rev. Mol. Cell Biol..

[105] Lourenco C., Resetca D., Redel C., Lin P., MacDonald A.S., Ciaccio R., Kenney T.M.G., Wei Y., Andrews D.W., Sunnerhagen M. (2021). MYC protein interactors in gene transcription and cancer. Nat. Rev. Cancer.

[106] Wang S., Zhang M., Zhang T., Deng J., Xia X., Fang X. (2020). microRNA-141 inhibits TGF-β1-induced epithelial-to-mesenchymal transition through inhibition of the TGF-β1/SMAD2 signalling pathway in endometriosis. Arch. Gynecol. Obstet..

[107] Peng X., Zhu Y., Wang T., Wang S., Sun J. (2023). Integrative analysis links autophagy to intrauterine adhesion and establishes autophagy-related circRNA-miRNA-mRNA regulatory network. Aging.

[108] Cobellis L., Caprio F., Trabucco E., Mastrogiacomo A., Coppola G., Manente L., Colacurci N., De Falco M., De Luca A. (2008). The pattern of expression of Notch protein members in normal and pathological endometrium. Am. J. Anat..

[109] Jiang N., Pan W., Li J., Cao T., Shen H. (2020). Upregulated circular RNA hsa_circ_0008433 regulates pathogenesis in endometriosis via miRNA. Reprod. Sci..

[110] Rossi J.J. (2008). Expression strategies for short hairpin RNA interference triggers. Hum. Gene Ther..

[111] Müller S., Appel B. (2017). In vitro circularization of RNA. RNA Biol..

[112] Ran F.A., Hsu P.D., Wright J., Agarwala V., Scott D.A., Zhang F. (2013). Genome engineering using the CRISPR-Cas9 system. Nat. Protoc..

[113] Zhou Q., Fang L., Tang Y., Wang Q., Tang X., Zhu L., Peng N., Wang B., Song W., Fu H. (2024). Exosome-mediated delivery of artificial circular RNAs for gene therapy of bladder cancer. J. Cancer.

[114] Shi X., Wang B., Feng X., Xu Y., Lu K., Sun M. (2020). circRNAs and exosomes: A mysterious frontier for human cancer. Mol. Ther.-Nucleic Acids.

[115] El-Andaloussi S., Lee Y., Lakhal-Littleton S., Li J., Seow Y., Gardiner C., Alvarez-Erviti L., Sargent I.L., Wood M.J.A. (2012). Exosome-mediated delivery of siRNA in vitro and in vivo. Nat. Protoc..

[116] Shtam T.A., Kovalev R.A., Varfolomeeva E.Y., Makarov E.M., Kil Y.V., Filatov M.V. (2013). Exosomes are natural carriers of exogenous siRNA to human cells in vitro. Cell Commun. Signal..

[117] Setten R.L., Rossi J.J., Han S.-P. (2019). The current state and future directions of RNAi-based therapeutics. Nat. Rev. Drug Discov..

